# Mining nematode protein secretomes to explain lifestyle and host specificity

**DOI:** 10.1371/journal.pntd.0009828

**Published:** 2021-09-29

**Authors:** Lucienne Tritten, Cristina Ballesteros, Robin Beech, Timothy G. Geary, Yovany Moreno

**Affiliations:** 1 Institute of Parasitology, University of Zurich, Zurich, Switzerland; 2 Institute of Parasitology, McGill University, Sainte-Anne-de-Bellevue, Canada; 3 School of Biological Sciences, Queen’s University Belfast, Belfast, United Kingdom; University of Liverpool, UNITED KINGDOM

## Abstract

Parasitic nematodes are highly successful pathogens, inflicting disease on humans, animals and plants. Despite great differences in their life cycles, host preference and transmission modes, these parasites share a common capacity to manipulate their host’s immune system. This is at least partly achieved through the release of excretory/secretory proteins, the most well-characterized component of nematode secretomes, that are comprised of functionally diverse molecules. In this work, we analyzed published protein secretomes of parasitic nematodes to identify common patterns as well as species-specific traits. The 20 selected organisms span 4 nematode clades, including plant pathogens, animal parasites, and the free-living species *Caenorhabditis elegans*. Transthyretin-like proteins were the only component common to all adult secretomes; many other protein classes overlapped across multiple datasets. The glycolytic enzymes aldolase and enolase were present in all parasitic species, but missing from *C*. *elegans*. Secretomes from larval stages showed less overlap between species. Although comparison of secretome composition across species and life-cycle stages is challenged by the use of different methods and depths of sequencing among studies, our workflow enabled the identification of conserved protein families and pinpointed elements that may have evolved as to enable parasitism. This strategy, extended to more secretomes, may be exploited to prioritize therapeutic targets in the future.

## Author summary

Parasitic helminths (worms) cause long-lasting infections. In order to survive in their hosts, this class of pathogens has developed various strategies; one of them consists of releasing soluble mediators (e.g., excretory/secretory (ES) proteins), which dampen the host immune response. Here, we analyzed and compared published ES protein catalogs of parasitic nematodes to identify common patterns as well as species-specific traits. Many proteins were common to multiple species, and a few were absent in secretions from the non-parasitic species *Caenorhabditis elegans*. This was the case of two glycolytic enzymes, aldolase and enolase, for which alternative functions have been proposed by others. A role in parasitic processes is intriguing. Our workflow enabled the identification of conserved protein families and pinpointed elements that may have evolved as to enable parasitism. This may prove useful in the future to identify and prioritize potential targets for therapeutic interventions.

## 1. Introduction

Nematodes are a ubiquitous and highly diverse group of organisms. A parasitic lifestyle has evolved at least 15 times in this phylum, with multiple species becoming reliant on plant or animal hosts for survival and reproduction [1].

Parasitic species cause debilitating pathologies and immense economic losses. In 2013, 1.75 billion humans were estimated to be infected with the major parasitic nematode species of the gastrointestinal (GI) tract (*Ascaris lumbricoides*, *Trichuris trichiura*, and the hookworms), mostly in tropical and subtropical areas of the world [2]. In Western countries, the economic impact of nematode parasites is huge, especially for livestock industries. GI nematodes alone cause production losses in up to 50% of ruminant farms analyzed in several European studies [3]. Plant parasitic nematodes cause projected yield losses of > 12% in crops worldwide, not only due to direct damage, but also by increasing the plants’ vulnerability to other microorganisms [4].

One peculiarity of nematode and other helminth infections is their typically long-lasting, chronic nature. In mammalian hosts, adult worms can live for several months to many years. For instance, filarial nematodes causing lymphatic filariasis (*Wuchereria bancrofti* and *Brugia* spp.) can persist for at least 6–8 years [5]. For *Onchocerca volvulus*, this can be 10–15 years (retrieved from https://www.cdc.gov/dpdx/onchocerciasis/index.html). Despite great differences in life cycles, preferential hosts and transmission modes, parasitic nematodes share a common capacity to manipulate their host’s immune system. Their immunomodulatory properties explain at least in part their longevity in mammalian hosts [6]. Hence, understanding the molecular dialogue between nematodes and their hosts that enables establishment of a chronic infection has raised great interest in the scientific community. A particularly attractive reason to understand the biological roles of parasite-derived molecules released into the host is that this menu may explain the basis for host-parasite specificity. It appears that the immune responses of mammals, for instance, are typically very capable of preventing establishment of nematodes following infection. The relatively few nematode species that establish successful infections in a given host must be able to modulate the immune response through this molecular language, which can be ‘understood’ by a small subset of potential hosts, but not by the majority of such species.

Nematode infections typically induce a regulatory, “Th2-type” immune response in mammals, involving CD4+ regulatory T cells, regulatory B cells, alternatively activated macrophages, and type 2 cytokines, among other effectors of the innate and adaptive arms of type 2 immunity [7,8]. A hallmark of chronic nematode infections is the achievement of nematode antigen-specific T cell unresponsiveness [7,8]. This type of immune evasion, called immunomodulation, is only achieved by live worms and involves different types of soluble mediators, released both passively and actively by the worm into its environment [7,9]. These mediators can be collected from cultivated live worms *in vitro*, and the so-called excretory/secretory (ES) products from a number of nematode species have been at least partially characterized biochemically. ES products are described as both substances that are actively secreted by helminths and products that are released during physiological processes such as digestion or egg-laying [9,10]. Hence, the composition of ES products can vary among different life-cycle stages [10]. ES products consist of different types of biomolecules, including proteins, peptides, small metabolites and RNA, many examples of which have immunomodulatory properties, often showing some redundancy in target and function [9,11]. Excretory/secretory proteins (ESP), termed the secretome, have been the most extensively studied component of nematode ES products and comprise functionally diverse categories of molecules, including chemokines, hormones, enzymes, antimicrobial peptides, etc. They are capable of (i) mimicking host molecules (e.g., homologues of mammalian cytokine macrophage migration inhibitory factor, MIF, which together with IL-4, induce alternative activation (suppressive profile) of macrophages) [12,13], (ii) inhibiting immunological processes (e.g., protease inhibitors such as cystatins impair antigen processing by antigen-processing cells and promote an immunosuppressive cytokine environment) [14], (iii) degrading key host molecules (e.g., acetylcholinesterase from GI nematodes can degrade the neurotransmitter acetylcholine, involved in fluid and mucus production in the gut) [9,15], or iv) facilitating entry into, and movement within, the host by destabilizing structural components (e.g., the plant-cell wall modifying enzymes of plant parasitic nematodes) [16].

The attractiveness of the immunomodulatory and anti-inflammatory properties of nematode ESP for drug or vaccine design has been acknowledged for many years. Promising vaccine candidates destined for use in humans or animals have been taken to early phases of clinical development [17]. For example, the Human Hookworm Vaccine Initiative has been assessing the use of recombinant *Necator americanus* ESP (e.g., Na-ASP-2, Na-GST-1, Na-APR-1), which have shown different degrees of success [18]. Similarly, the anti-inflammatory properties of the ES-62 glycoprotein from the filarial parasite *Acanthocheilonema viteae*, which are mediated by post-translational modification of this protein with phosphorylcholine, may lead to treatments for various inflammatory conditions [19–22].

In this study, we gathered published protein secretomes of multiple species of parasitic nematodes to identify common patterns as well as species-specific protein secretion traits. These organisms span 4 nematode clades, including 3 plant and 16 animal parasites. Among parasites of mammals, 12 occupy the GI tract of their hosts, one is located in blood vessels, one resides in lymph nodes, and 2 inhabit other tissues. They display a wide variety of preferential hosts and life histories. Finally, we included previously unpublished secretome data from the non-parasitic free-living nematode *Caenorhabditis elegans* to highlight ESP components that may be associated with parasitism.

## 2. Methods

### 2.1. *C*. *elegans* ES protein characterization by LC-MS/MS

*Caenorhabditis elegans* N2 strain was maintained under standard conditions on agar plates with *Escherichia coli* OP50 as food source [23]. L4 –young adult stage worms from a synchronized culture were recovered from the plates and washed extensively in M9 buffer to eliminate bacteria and other debris that could interfere with the downstream analysis. The worms were then incubated in M9 buffer for 4 h and ESP collected by pelleting the worms by centrifugation at 1150 x *g* for 2 min. A cocktail of protease inhibitors (Sigma No P8849, St. Louis, MO) was added to the recovered media, which was then passed through a 0.22 μm filter. Sample concentration and further processing was performed as previously described [24]. ESP mixtures were separated by SDS-PAGE on a 7–15% gradient acrylamide gel, followed by staining with Coomassie Brilliant Blue G. All procedures were performed at Genome Quebec as previously described, including LC-MS/MS [24]. The entire lane was subjected to automated band excision to generate 13 contiguous bands which were subjected to reduction, cysteine-alkylation, and in-gel tryptic digestion in a MassPrep Workstation (Micromass, Manchester, UK). Twenty μl of the tryptic digest solution were injected on a Zorbax 300SB-C18 pre-column (5 × 0.3 mm, 5 μm) equilibrated with water containing acetonitrile (5%) and formic acid (0.1%) using the Micro Well-plate sampler and the IsoPump modules of an Agilent 1100 Series Nanoflow HPLC. Following washing for 5 min at 15 μl/min, the pre-column was back-flushed to a 75 μm i.d. PicoFrit column (New Objective, Woburn, MA) filled with 10 cm BioBasic C18 packing (5 μm, 300 Å) by the acetonitrile gradient supplied by the Agilent series 1100 Nanopump to elute the peptides toward the mass spectrometer at a flow rate of 200 ηl/min. Eluted peptides were analyzed in a Q-TOF micro (Waters Micromass, Manchester, UK) equipped with a Nanosource modified with a nanospray adapter (New Objective, Woburn, MA). The MS survey scan was set to 1 s (0.1 s interscan) and recorded from 350 to 1,600 *m/z*. MS/MS scans were acquired from 50 to 1,990 *m/z*, scan time was 1.35 s, and the interscan interval was 0.15 s. Doubly and triply charged ions were selected for fragmentation with collision energies calculated using a linear curve from reference collision energies.

MS raw data from a single run were acquired on the Data Directed Analysis feature in the MassLynx (Micromass) software with a 1, 2, 4 duty cycle (1 sec in MS mode, 2 peptides selected for fragmentation, maximum of 4 sec in MS/MS acquisition mode). MS/MS raw data were transferred from the Q-TOF Microcomputer to a server and automatically manipulated for generation of peaklists by employing Distiller version 2.3.2.0 (http://www.matrixscience.com/distiller.html) with peak picking parameters set at 5 for Signal Noise Ratio (SNR) and at 0.4 for Correlation Threshold (CT). Briefly, MS/MS peak lists (MGF files) generated were analyzed using Mascot (Matrix Science, London, UK) and X! Tandem (Version: 2007.01.01.1) [25,26]. Scaffold (version 4.7.3, Proteome Software Inc., Portland, OR) was used to validate MS/MS based peptide and protein identifications [27]. Peptide identifications were accepted if established at > 95.0% probability and protein identifications were accepted if established at > 95.0% probability and contained at least 2 identified peptides. Proteins that contained similar peptides and could not be differentiated based on MS/MS analysis alone were grouped to satisfy the principles of parsimony. Searches were performed in a database set consisting of *C*. *elegans* and *Escherichia coli* proteins in the Uniprot database (Taxon IDs: 6239 and 562, release 2018_04). To rank the proteins based on absolute abundance, the emPAI spectrum counting method was employed in Scaffold [28]. Data from the 13 files corresponding to 13 gel pieces were analyzed in Mudpit [29] to generate a single report. The resulting protein list was further analyzed by Blast2GO [30,31] and SignalP 5.0 [32]. The data was deposited on Mendeley Data (doi:10.17632/727hjmhwn5.1; https://data.mendeley.com/datasets/727hjmhwn5/1).

### 2.2. Selection of nematode protein secretomes

**Fig 1:**
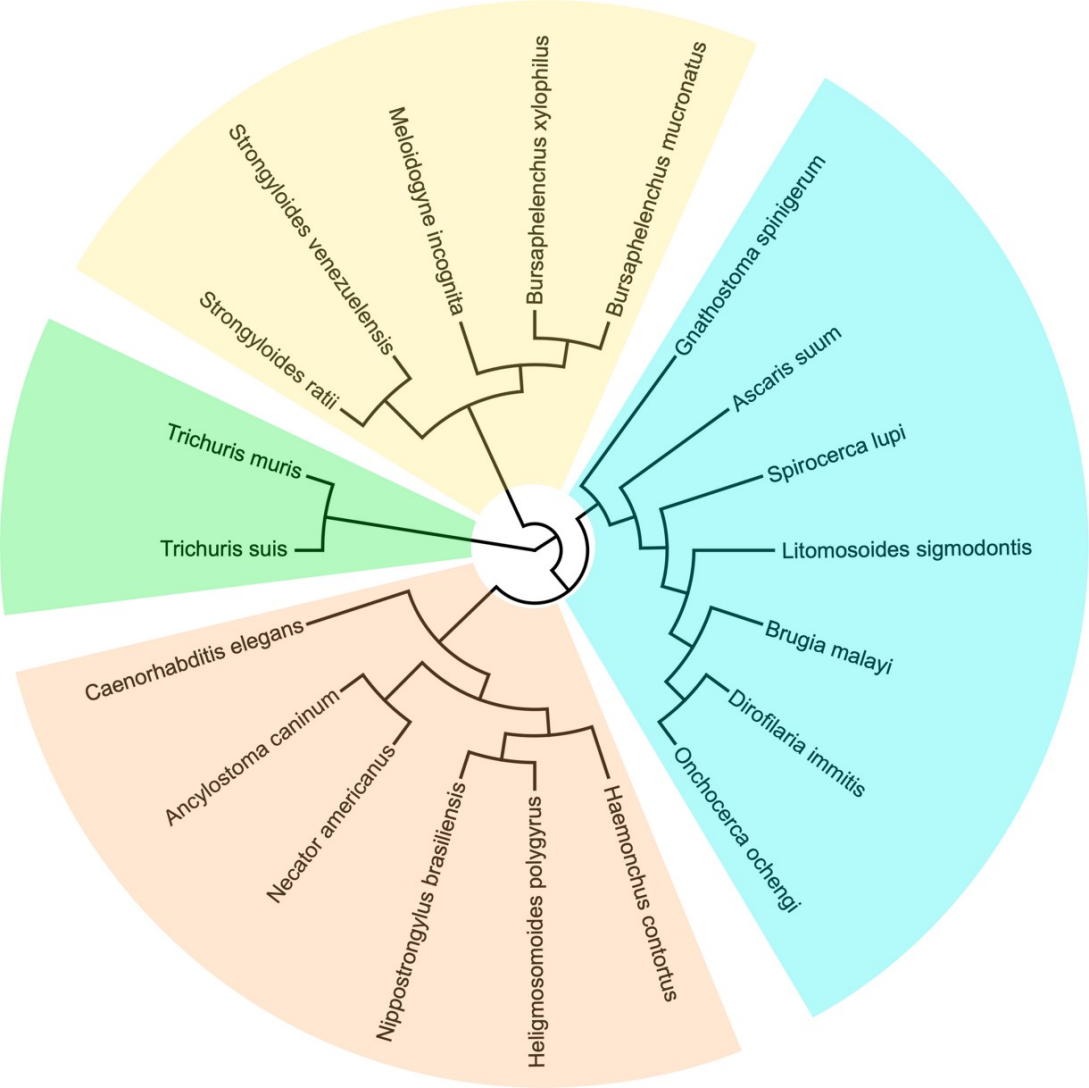
Helminth phylogeny. The 20 selected species are placed along the nematode clades containing parasitic species according to recent phylogenetic analyses [34,61,62]. *Trichuris* spp. populate clade I (green); clade III (blue) contains the filarial nematodes, *S*. *lupi*, *A*. *suum*, and *G*. *spinigerum*; clade IV (yellow) contains *Strongyloides* spp. and the selected plant parasites; clade V (orange) contains *C*. *elegans* and other Rhabditomorpha.

We selected all published parasitic nematode whole ESP catalogues published before May 2020 and produced experimentally by MS (Fig 1 and Table 1) using an arbitrary cutoff of 20 unique proteins (or sequence descriptions), for which accession numbers were publicly accessible, and analyzed them between August 2019 and May 2020. To identify reports of nematode secretomes, we searched PubMed using the following terms: "nematode secreted proteins proteomic", "nematode secretome", "nematode excretory/secretory". Proteins found in extracellular vesicle (EV) preparations have not been taken into account, as they have been the subject of a recent review [33]. We identified secretomes from species in all five major clades in the phylum Nematoda, with several independent transitions from free-living to vertebrate parasitism [34,35]. We included secretomes of *Haemonchus contortus* [36,37], *Ancylostoma caninum* [38,39], *Heligmosomoides polygyrus bakeri* [24,40], *Nippostrongylus brasiliensis* [41], *Necator americanus* [42], *Strongyloides ratti* [43], *Strongyloides venezuelensis* [44], *Ascaris suum* [45–47], *Dirofilaria immitis* [48,49], *Brugia malayi* [50–52], *Onchocerca ochengi* [53], *Litomosoides sigmodontis* [54], *Spirocerca lupi* [55], *Gnathostoma spinigerum* [56], *Trichuris muris* [57], and *Trichuris suis* [58]. Plant parasitic species included *Meloidogyne incognita* [16], *Bursaphelenchus xylophilus* [59,60], and *Bursaphelenchus mucronatus* [59]. As a non-parasitic comparator, we included our previously unpublished ES data from *Caenorhabditis elegans*. As noted in Table 1, various life-cycle stages of the parasites were included; overall, we analyzed 18 adult secretomes and 12 larval secretomes.

**Table 1:** Technical properties of the selected studies. Twenty-six studies, covering 20 parasite species, describing ESP analyzed in mass spectrometry experiments were considered. Methodological details are noted, as well as the number of unique proteins used from each report.

Species	Main definitive hosts	Source	Stage/sex	Total number of worms	ID or 2D PAGE?	Spot/band selections	MS platform	Unique proteins accessions available for the present work	Reported proportion with a signal peptide
ADULTS									
***C*. *elegans***	-	Current work	L4-young adults (hermaphrodites)	N/A	1D	Whole lane	Q-TOF micro (Waters Micromass)	183	93.5%
***H*. *contortus***	Sheep, goat	[36]	Mixed sexes	N/A	2D	130 spots	Voyager DE-STR MALDI-TOF mass spectrometer (Applied Biosystems), ESI-Q-TOF (Micromass Ltd)	33	-
***H*. *contortus***	Sheep, goat	[37]	Females and males	N/A	No	-	Q Exactive Plus mass spectrometer (Thermo Scientific)	621	54.1%
***A*. *caninum***	Canids, felids	[38]	Mixed sexes	N/A	OFFGEL electrophoresis	no	micrOTOF-Q (Bruker)	90	57%
***A*. *caninum***	Canids, felids	[39]	Mixed sexes	300	OFFGEL electrophoresis	18 bands	AB SCIEX Triple TOF+ 5600 mass spectrometer (Applied Biosystems)	315	-
***H*. *p*. *bakeri***	Rodents	[24]	Mixed sexes	N/A	1D	Whole lane	Q-TOF micro (Waters Micromass)	20	-
***H*. *p*. *bakeri***	Rodents	[40]	Mixed sexes	N/A	1D	N/A	Hybrid LTQ-Orbitrap XL instrument (Thermo Fisher)	361	-
***N*. *brasiliensis***	Rodents	[41]	Mixed sexes	N/A	2D and OFFGEL electrophoresis	no	AB SCIEX Triple TOF+ 5600 mass spectrometer (Applied Biosystems)	162	43.8% *
***N*. *americanus***	Human (maintained in hamsters)	[42]	Mixed sexes	N/A	OFFGEL electrophoresis	N/A	AB SCIEX Triple TOF+ 5600 mass spectrometer (Applied Biosystems)	198	48%
***S*. *ratti***	Rat	[43]	Females	N/A	1D	Whole lanes	LTQ linear ion trap mass spectrometer (Thermo Scientific)	219	27.9%
***S*. *venezuelensis***	Rat	[44]	Females	N/A	1D	Whole lanes	LCMS-IT-TOF mass spectrometer (Shimadzu Scientific Instruments)	154	20.1% *
***A*. *suum***	Pig	[45]	Females	10 adult females	1D	Whole lane	LTQ-Orbitrap Elite mass spectrometer (Thermo Fisher)	175	40%
***A*. *suum***	Pig	[46]	Mixed adults	NA	No	-	Q Exactive mass spectrometer HF (Thermo Fisher Scientific)	101	-
***D*. *immitis***	Canids, felids	[48]	Mixed adults	56 worms	1D	Whole lane	LTQ-FT Ultra mass spectrometer (Thermo Fisher)	102	20.9%
***D*. *immitis***	Canids, felids	[49]	Adult males and females	40 males, 40 females	No	-	AB Sciex Triple TOF+ 5600 mass spectrometer (Applied Biosystems)	84	-
***B*. *malayi***	Human	[52]	Adult females and males	N/A	1D	Whole lanes	Q-TOF micro (Waters Micromass)	194	27.2%
***B*. *malayi***	Human	[50]	Adult females and males	NA	No	-	LIT-FT mass spectrometer	375	15% *
***B*. *malayi***	Human	[51]	Mixed adults	50 worms	2D and no electrophoresis	23 spots	MALDI-ToF/ToF and LC-MS/MS (Proteomics Analyzer 4700 (Applied Biosystems)	77	42.3%
***O*. *ochengi***	Cattle	[53]	Adults (from nodule fluid)	N/A	No	-	LTQ-Orbitrap Velos and Q-Exactive mass spectrometers (both Thermo Fisher Scientific)	94	50%
***L*. *sigmodontis***	Rodents	[54]	Adult males, pre-gravid adult females, gravid adult females	N/A	No	-	LTQ-Orbitrap Velos (Thermo Scientific)	297	31.1% *
***S*. *lupi***	Canids, felids	[55]	Adult females and males	N/A	No	-	Q Exactive Plus (Thermo Scientific)	128	-
***T*. *muris***	Mouse	[57]	Mixed adults	N/A	No	-	5600 mass spectrometer (AB Sciex)	145	41.9%
***T*. *suis***	Pig	[58]	Mixed adults and 28 days old larvae	N/A	No	-	5600 mass spectrometer (AB Sciex)	342	26% *
**LARVAL STAGES**									
***H*. *contortus***	Sheep, goat	[37]	L3 and L4	N/A	No	-	Q Exactive Plus mass spectrometer (Thermo Scientific)	307 and 527	58.2% (L3), 53.3% (L4)
***N*. *brasiliensis***	Rodents	[41]	L3	N/A	OFFGEL electrophoresis	no	AB Ssciex Triple TOF+ 5600 mass spectrometer (Applied Biosystems)	31	43.8% *
***M*. *incognita***	Plants	[16]	Juvenile infective stage 2	N/A	No	-	linear ion trap tandem mass spectrometers (LTQ, Thermo Electron Corporation, San Jose, CA)	472	-
***S*. *ratti***	Rat	[43]	L3	N/A	1D	Whole lanes	LTQ linear ion trap mass spectrometer (Thermo Scientific)	336	22.3%
***S*. *venezuelensis***	Rat	[44]	L3	N/A	1D	Whole lanes	LCMS-IT-TOF mass spectrometer (Shimadzu Scientific Instruments)	415	20.1% *
***A*. *suum***	Pig	[47]	L3- egg, L3-lungs, L4	N/A	1D	Whole lane	ESI-Q-TOF Premier (Waters)	20, 45, and 58	62% *
***B*. *malayi***	Human	[50]	Advanced (molting) L3	N/A	No	-	LIT-FT mass spectrometer	27	15% *
***G*. *spinigerum***	Canids, felids, (human)	[56]	Advanced L3	N/A	1D	Whole lanes	nanoLC-MS/MS; micrOTOF-Q II (Bruker)	21	-
***L*. *sigmodontis***	Rodents	[54]	L3	N/A	No	-	LTQ-Orbitrap Velos (Thermo Scientific)	20	31.1%
***S*. *lupi***	Canids, felids	[55]	L3 and L4 females	N/A	No	-	Q Exactive Plus (Thermo Scientific)	117 and 131	-
***T*. *suis***	Pig	[58]	Early larvae (up to 21 days)	N/A	No		5600 mass spectrometer (ABSciex)	94	26% *
**MIXED STAGES**									
***B*. *xylophilus***	Plants	[60]	All stages	N/A	No	-	LTQ Velos orbitrap mass spectrometer (Thermo Fisher Scientific)	1501	41.3%
***B*. *xylophilus***	Plants	[59]	All stages	N/A	No	-	Triple TOF 5600 (AB Sciex)	495	-
***B*. *mucronatus***	Plants	[59]	All stages	N/A	No	-	Triple TOF 5600 (AB Sciex)	326	-

N/A: not available

* based on proteins from several stages confounded.

### 2.3. Data analysis

Comparative secretome analysis among ecologically diverse species may help prioritize proteins that play essential roles in parasitism for functional studies and may contribute to our understanding of the molecular basis of host-parasite specificity. To do so, protein families and orthology analyses allowed us to re-analyze all collected data simultaneously with the same tool, and resulted in a sequence-based clustering, independent of initial annotation.

#### 2.3.1. Analysis of protein families

Description of the proteins based on the Pfam families and domains was performed using Pfam_scan.pl, a Perl script calling HMMER v3 to search a FASTA file against a library of Pfam HMMs [63]. The gathering bit score (—cut_ga) threshold is employed as default parameter. Unique Pfam accessions (counted only once even if it appeared several times in a dataset) were used to address common and unique features across species (reports pooled regardless of the life-cycle stage or methodological differences). ESPs from adult worms and larval stages were analyzed separately, including *B*. *mucronatus* and *B*. *xylophilus* in both analyses, as these datasets comprised mixed stages.

#### 2.3.2. Orthology inference analysis

Protein sequences from each dataset were aligned against each other (in a blast all-vs-all) using OrthoFinder [64–67] with default parameters. The program finds orthologs and orthogroups and infers gene and species trees. It was fed with adult ESP sequences (fasta), pooling data from different studies for each species: *A*. *caninum* (n = 405), *A*. *suum* (n = 277), *B*. *malayi* (n = 646), *B*. *mucronatus* (n = 326), *B*. *xylophilus* (n = 1996), *C*. *elegans* (n = 184), *D*. *immitis* (n = 180), *H*. *contortus* (n = 653), *H*. *polygyrus* (n = 381), *L*. *sigmodontis* (n = 297), *N*. *americanus* (n = 198), *N*. *brasiliensis* (n = 162), *O*. *ochengi* (n = 94), *S*. *lupi* (n = 128), *S*. *ratti* (n = 220), *S*. *venezuelensis* (n = 154), *T*. *muris* (n = 145), *T*. *suis* (n = 342). The workflow comprises a DIAMOND BLAST [68] all-versus-all to assign proteins to orthogroups. It then produces a distance matrix (using FastME [69]) to finally generate gene- and species-level trees. ESPs from adult worms and larval stages were analyzed separately; *B*. *mucronatus* and *B*. *xylophilus* were included in both analyses, as these datasets comprised mixed stages. Similarly, the program was run with pooled data from all available larval stages (and from different studies, excluding microfilariae) for each species: *A*. *suum* (n = 120), *B*. *malayi* (n = 27), *B*. *mucronatus* (n = 326), *B*. *xylophilus* (n = 1996), *G*. *spinigerum* (n = 21), *H*. *contortus* (n = 834), *L*. *sigmodontis* (n = 20), *M*. *incognita* (n = 472), *N*. *brasiliensis* (n = 31), *S*. *lupi* (n = 248), *S*. *ratti* (n = 334), *S*. *venezuelensis* (n = 415), *T*. *suis* (n = 94). Resulting data were deposited in Mendeley Data (DOI: 10.17632/2skvdrf3rt.1; https://data.mendeley.com/datasets/2skvdrf3rt/1).

#### 2.3.3. Phylogeny

Protein sequences were aligned using MAFFT [70]. Identical sequences were removed to leave only a single representative. Positions containing more than 10% gaps were excluded. A maximum likelihood phylogeny was inferred with branch support evaluated by SH-like statistics (PhyML [71]). Figures were produced using the iTOL online interface [72].

## 3. Results

### 3.1. *C*. *elegans* secretome

MS analysis of *C*. *elegans* ESP led to the identification of 184 proteins. Table 2 shows the 10 most abundant proteins; all proteins identified are shown in S1 Table. The 3 most common biological processes were ‘innate immune response’ (GO:0045087, 43/184 proteins), ‘defense response to Gram-positive bacterium’ (GO:0050830, 20/184 proteins), and ‘defense response to Gram-negative bacterium’ (GO:0050829, 19/184 proteins). The 3 most common molecular functions were ‘carbohydrate binding’ (GO:0030246, 18/184 proteins), ‘aspartic-type endopeptidase activity’ (GO:0004190, 10/184 proteins), and ‘serine-type carboxypeptidase activity’ (GO:0004185, 9/184 proteins). Finally, the 3 most common cell compartments were ‘integral component of membrane’ (GO:0016021, 22/184 proteins), ‘membrane raft’ (GO:0045121, 18/184 proteins), and ‘extracellular space’ (GO:0005615, 16/184 proteins). The large majority of *C*. *elegans* ESP were predicted to have a signal peptide, with 171 proteins presenting a putative cleavage site.

**Table 2:** Top 10 proteins identified in the *C*. *elegans* secretome. Proteins were ranked by relative abundance using the emPAI values in Scaffold.

Abundance Ranking	Uniprot ID	Protein Description	Quantitative Value (Normalized emPAI)
1	Q9TSVS4	Aspartic protease 1	230
2	Q19698	Invertebrate lysozyme protein 5, isoform a	183
3	P34528	Putative serine protease K12H4.7	109
4	Q20219/ A0A131MBU3	Protein irg-7 (infection response gene 7)	85
5	Q21152	Fatty acid/retinol binding protein	74
6	G5ECR0	Lectin C-type domain protein	41
7	O45444	C-type lectin	38
8	Q19853	Protein irg-7	37
9	O01530	Aspartic protease 6	34
10	Q94246	GEI-4 (Four) interacting protein	30

### 3.2. Protein families

Representatives of the transthyretin-like protein family (PF01060.23) were found in ESP from adult worms of all 18 species included in this analysis, while cyclophilin-type peptidyl-prolyl cis-trans isomerase (PF00160.21) was present in all adult secretomes except *N*. *americanus*. Stratification by species (upon pooling datasets for the same species) identified 5 Pfam accessions common to all parasitic species examined but absent in *C*. *elegans*. Two of them were enolase (PF03952.16 and PF00113.22); the other 3 were fructose-bisphosphate aldolase (PF00274.19), C-terminal domain of 1-Cys peroxiredoxin (PF10417.9), and alkyl hydroperoxide reductase (AhpC)/thiol specific antioxidant (TSA) family (PF00578.21). Thirty-nine Pfam accessions were unique to the plant parasites (belonging to the same genus, both describing ESPs from mixed stages), but no Pfam was specifically associated with animal parasites. No Pfam was found to be clade-specific, although 2 accessions were unique to *T*. *muris* and *T*. *suis* (clade I, same genus): C8 (PF08742.11) and calponin homology (PF18383.1) domains. Similarly, no pattern was discernable with host localization (GI tract vs other tissues). Detailed Pfam accessions are provided in S2 Table.

Clustering by species yielded 13 datasets for larvae (all larval stages confounded) for comparison. No Pfam accession was common to all. Cyclophilin-type peptidyl-prolyl cis-trans isomerase (PF00160.21) was found in larval secretions from all species except *G*. *spinigerum* (see S2 Table). Forty-seven accessions were common (and unique) to all 3 plant parasites. Three Pfams were uniquely found in clade IV larvae (*B*. *mucronatus*, *B*. *xylophilus*, *M*. *incognita*, *S*. *ratti* and *S*. *venezuelensis*): aconitase (PF00330.20), aconitase C-terminal domain (PF00694.19), and lipocalin (PF00061.23). Apyrase (PF06079.11) was common to larvae of the two clade V parasites examined (*H*. *contortus* and *N*. *brasiliensis*).

### 3.4. Orthology analysis

**Fig 2:**
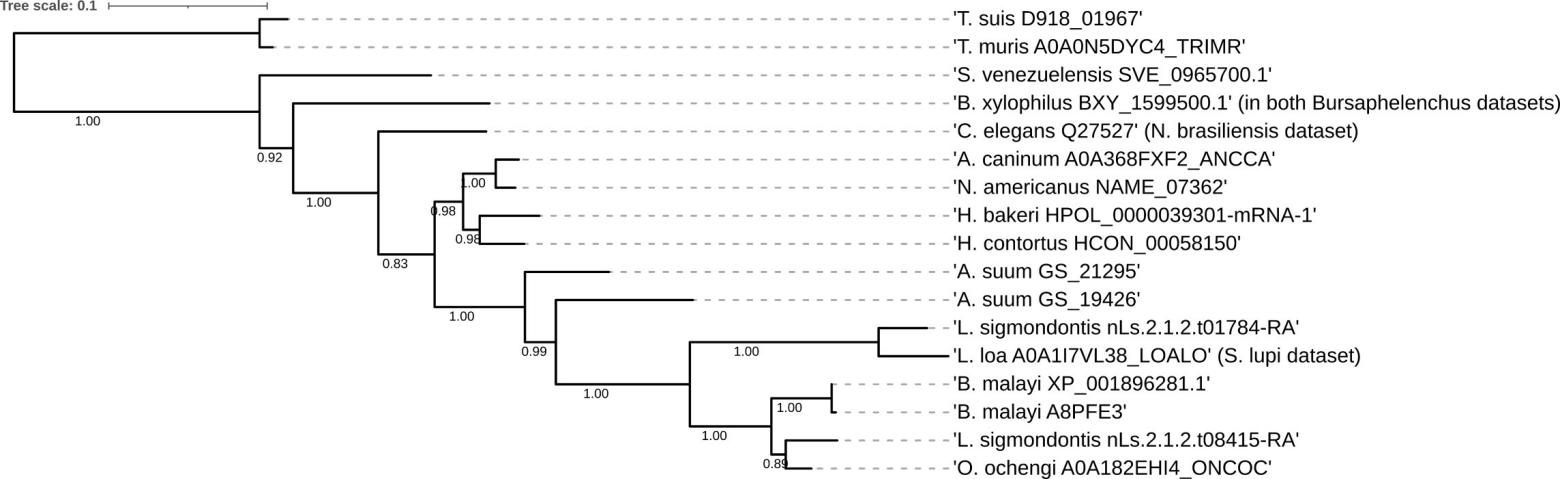
Maximum likelihood phylogeny of enolase protein sequences. Scale bar represents distance. Numbers indicate the SH-like support for each node. The tree is rooted at the divergence of clade I nematodes. The tree follows the expected divergence based on species. The dataset of origin is indicated in brackets, in case a homologous species name was used as description in the original reports. The placement of *C*. *elegans* Q27527 (sequence from the *N*. *brasiliensis* dataset) outside clade V does not have reliable support in the data (bootstrap = 0.83). *D*. *immitis* and *S*. *ratti* also showed enolase sequences, which are not depicted in this graph due to sequence selection described in the methods (2.3.3.).

**Fig 3:**
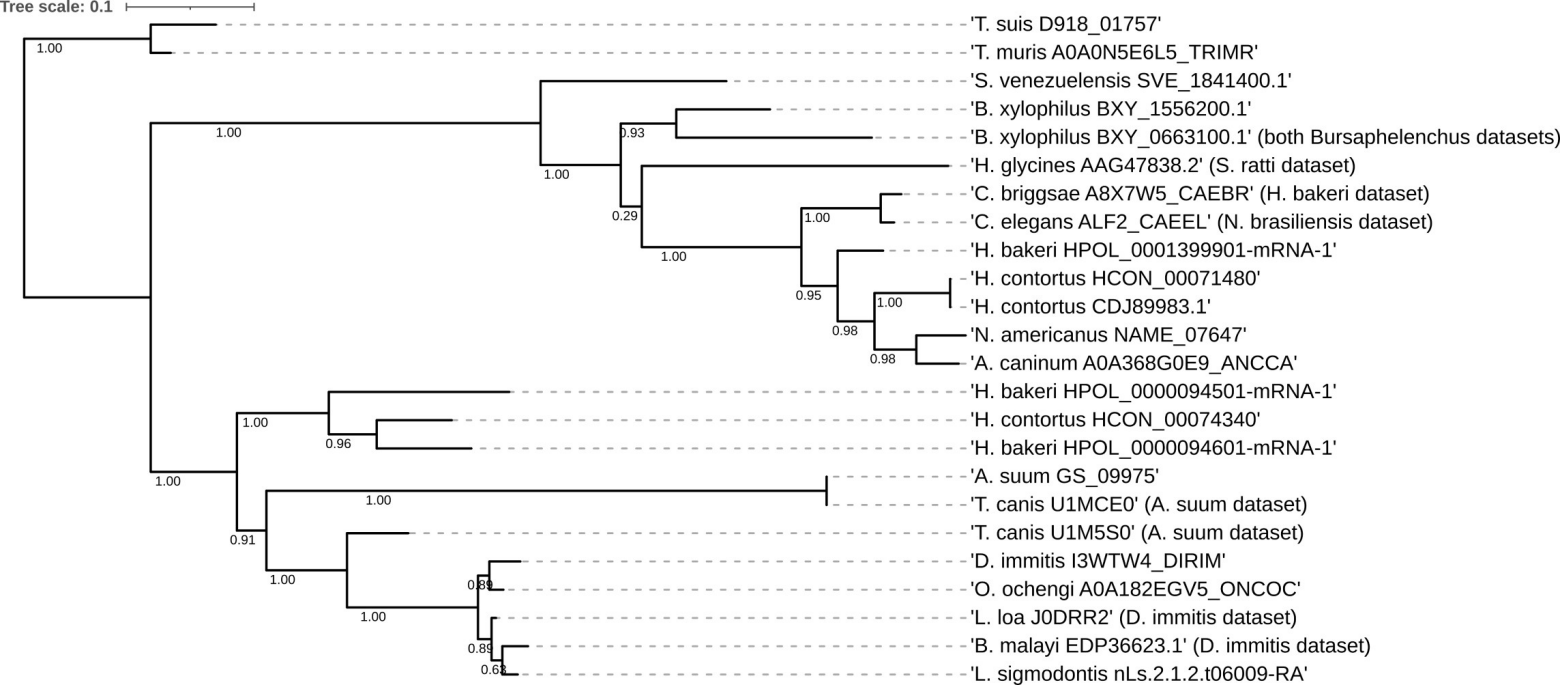
Maximum likelihood phylogeny of aldolase protein sequences. Scale bar represents distance. Numbers indicate the SH-like support for each node. The tree is rooted at the divergence of clade I nematodes. The tree follows the expected divergence based on species. The presence of multiple sequences from a species at different points in the tree indicates the presence of gene duplication. The aldolase orthogroup is likely represented by two genes, which form two groups; within each group, the expected phylogeny is respected. *B*. *malayi* and *S*. *lupi* also showed aldolase sequences, which are not depicted in this graph due to sequence selection described in the methods (2.3.3.).

Using adult ESP, OrthoFinder assigned 5783 genes (85.2% of total) to 905 orthogroups. An orthogroup is a group of genes descended from a single gene in the last common ancestor of a group of species and is identified from a blast all-versus-all analysis [64,65]. *B*. *malayi* (from 3 individual reports) was the species with the fewest assigned genes (66.3%); ≥ 79% of ESP were assigned to orthogroups for all other species. A fraction of proteins could not be assigned to orthogroups, due to i) protein sampling (ESP catalogs and not whole proteomes) and ii) varying and relatively small dataset sizes. Maximum likelihood phylogenies of 10 highly represented orthogroups of interest (OG0000000: transthyretin-like family; OG0000006: peptidyl-prolyl cis-trans isomerase; OG0000025: enolase; OG0000020: aldolase; OG0000010: fatty-acid and retinol-binding protein 1; OG0000027: peroxiredoxin/AhpC/TSA family; OG0000004: galectin; OG0000034: 14-3-3 protein; OG0000064: nucleoside diphosphate kinase; OG0000022: actin) and comparison with the species tree based on the ParaSite database [34] did not identify any clades inconsistent with the expected species relationship. Figs 2 and 3 show representative phylogenies of the enolase (OG0000025) and aldolase sequences OG0000020 [34]. Details of resulting orthogroups can be found in Mendeley data (DOI: 10.17632/2skvdrf3rt.1; https://data.mendeley.com/datasets/2skvdrf3rt/1).

The orthogroup corresponding to transthyretin-like proteins was found consistently in all adult ESP (18 species; Table 3). Fructose-bisphosphate aldolase and enolase were represented in ESP of all parasite species but not in secretions from *C*. *elegans*. Peptidyl-prolyl cis-trans isomerase was found in all datasets except *N*. *americanus*, whereas fatty-acid and retinol-binding protein 1 was missing in both *Trichuris* spp. (clade I species) only. As many as 19 orthogroups appeared unique to *Trichuris* spp. (clade I), and only one was found to be unique to the four clade IV species (OG0000377, which corresponds to a 4-hydroxyphenylpyruvate dioxygenase upon BLAST search). No orthogroup was unique and specific to clade III or V nematodes. Plant parasites (*Bursaphelenchus* spp.) shared 100 unique orthogroups. Localization in the host did not reveal further patterns. Orthogroups containing 14-3-3 protein (OG0000034), peroxiredoxin/AhpC/TSA family (OG0000027), and galectin (OG0000004), were detected in 16/18 species. Nucleoside diphosphate kinase (OG0000064), actin (OG0000022), malate dehydrogenase (OG0000041), triosephospate isomerase (OG0000044), glutathione S-transferase (OG0000009), protein disulfide isomerase (OG0000012), and glyceraldehyde 3-phosphate dehydrogenase (OG0000053) were detected in 15/18 species.

**Table 3:** Most common ESPs based on orthology analysis. ESPs were assigned to orthogroups in OrthoFinder. The number of species where at least one protein was assigned to a given orthogroup is indicated, along with the species where the orthogroup was found to be absent.

Number of species	Orthogroup	Protein name/function	Absent in ES from
18/18	OG0000000	Transthyretins	*-*
17/18	OG0000020	Fructose-bisphosphate aldolase	*C*. *elegans*
	OG0000025	Enolase	
	OG0000006	Peptidyl-prolyl cis-trans isomerase	*N*. *americanus*
16/18	OG0000010	Fatty-acid and retinol-binding protein 1	*T*. *muris*, *T*. *suis*
	OG0000027	Peroxiredoxin/AhpC/TSA family	*C*. *elegans*, *S*. *lupi*
	OG0000004	Galectin	*C*. *elegans*, *O*. *ochengi*
	OG0000034	14-3-3 protein	*A*. *suum*, *N*. *americanus*
15/18	OG0000064	Nucleoside diphosphate kinase	*N*. *americanus*, *O*. *ochengi and T*. *muris*
	OG0000022	Actin	*C*. *elegans*, *O*. *ochengi*, *S*. *lupi*
	OG0000041	Malate dehydrogenase	
	OG0000044	Triosephosphate isomerase	*B*. *mucronatus*, *C*. *elegans*, *N*. *americanus*
	OG0000009	Glutathione S-transferase	*B*. *malayi*, *C*. *elegans*, *S*. *venezelensis*
	OG0000012	Protein disulfide-isomerase	*A*. *suum*, *C*. *elegans*, *O*. *ochengi*
	OG0000053	Glyceraldehyde-3-phosphate dehydrogenase	*A*. *caninum*, *N*. *brasiliensis*, *S*. *ratti*
14/18	OG0000024	Superoxide dismutase [Cu-Zn]	*H*. *polygyrus*, *O*. *ochengi*, *S*. *venzuelensis*, *T*. *muris*
	OG0000003	Cysteine protease	*D*. *immitis*, *S*. *ratti*, *S*. *venezuelensis*, *T*. *muris*
	OG0000002	Aspartic protease	*D*. *immitis*, *S*. *lupi*, *S*. *venezuelensis*, *T*. *suis*
	OG0000018	Kunitz/Bovine pancreatic trypsin inhibitor domain containing protein	*D*. *immitis*, *S*. *lupi*, *S*. *ratti*, *T*. *muris*
	OG0000013	Major sperm protein	*C*. *elegans*, *N*. *americanus*, *S*. *lupi*, *S*. *venezuelensis*
	OG0000049	Phosphoenolpyruvate carboxykinase	*B*. *mucronatus*, *C*. *elegans*, *D*. *immitis*, *S*. *venezuelensis*
	OG0000032	Chitin binding Peritrophin-A domain protein	*B*. *malayi*, *C*. *elegans*, *S*. *lupi*, *T*. *muris*
	OG0000029	Heat shock 70 kDa protein	*A*. *suum*, *C*. *elegans*, *O*. *ochengi*, *S*. *venezuelensis*

Between 1 (*D*. *immitis*) and 105 (*B*. *xylophilus*) species-specific orthogroups were detected in all species except *B*. *mucronatus*, for which no orthogroup was found to be unique to this species.

ESP from larval stages were available for 13 species. The number of orthogroups varied more substantially among larval datasets: only 13 orthogroups were found for *G*. *spinigerum*, but 671 for *B*. *xylophilus*. No orthogroup was present in all species. The most common, represented by cyclophilin-type peptidyl-prolyl cis-trans isomerase, was found in all larval secretomes except *G*. *spinigerum*. Common to 11 species were transthyretin-like protein and galectin. Other commonly represented proteins included actin, aldolase, serpin, nucleoside diphosphate kinase, and major allergen protein, among others. Larval datasets included three plant parasites, including *M*. *incognita*. Together with the *Bursaphelenchus* spp., they shared 41 unique orthogroups, of which 41% were ribosomal proteins.

### 3.5. A closer look at aldolase and enolase

Present in all secretomes except of the nonparasitic *C*. *elegans*, we examined our data on aldolase and enolase. OrthoFinder produces resolved gene trees by conducting a duplication-loss-coalescence analysis and identifies the more parsimonious interpretation of the tree, as well as putative gene duplication events. As such, 13 and 9 duplication events were detected for aldoalse and enolase, respectively. The maximum parsimony phylogenies for enolase and aldolase (computed with a different dataset) are shown in Figs 2 and 3, respectively. Both trees follow the expected divergence based on species [34]; some gene duplication events are confirmed with this method. The aldolase orthogroup is likely represented by two genes, which form two groups; within each group, the expected phylogeny is respected.

## 4. Discussion

Nematode parasite ESP are distinct from the set of somatic proteins, in class and/or abundance [73]. Many of these proteins seem to be involved in nutrient uptake and transport, reflecting the strategies employed by these parasites to capture and exploit energy sources from the host, necessary for establishing a chronic infection. Much attention has been paid to their involvement in immune evasion/modulation, preventing elimination and development of host immunity. Secretomes may provide valuable information on the molecules and pathways involved in these events. In fact, helminth ES products represent an anti-inflammatory pharmacopeia that eventually may be exploited to treat autoimmune and inflammatory diseases [74].

This work presents the first comprehensive comparison of the protein components of nematode ESP from 20 species (18 species for adult secretomes and 12 species for mixed larval secretomes) based on experimentally-generated samples. Our results are in overall qualitative agreement with a similar study, based on 31 nematode secretomes predicted *in silico* [75]. In this prior study, secreted proteins were slected based on the presence of a signal peptide; however, many parasite ESP—especially those contained in EVs–lack these sequences. We compared the results of studies that varied in methodology and secretome coverage depth (amount of protein analyzed and false discovery rate cutoffs); hence, in the number of proteins detected, which may affect the degree of similarity between datasets. We also acknowledge the challenges represented by the complex sources of the 18 adult and 12 larval secretomes, sometimes comprising mixed stages, and representing many anatomical sources of protein secretion, including uterine fluid, and spanning infective as well as free-living stages. The number of worms, conditions, and duration of *in vitro* cultivation used to obtain the preparations analyzed in this study varied as well. Culture conditions are expected not to reflect accurately the *in vivo* situation. Similarly, maintenance of *C*. *elegans* on agar plates is an artificial situation; standardized *E*. *coli* cultures do not belong in the nematode’s diet in the wild [76]. Thus, laboratory settings may have an impact on the secretomes analyzed here and elsewhere, but the extent of it remains unknown. For instance, the fact that *C*. *elegans* ESP contribute heavily to defense mechanisms against Gram-negative bacteria (such as *E*. *coli*) might be explained by culture conditions. A further limitation of this comparative work was only one secretome from a non-parasitic species fulfilled our selection criteria. Hence, the current analysis of traits of parasitism is preliminary and will require validation with additional species. Nonetheless, our work provides a functional dimension (strictly qualitative, ignoring redundancies and abundance) to these ESP catalogs, and common trends and cluster-specific differences have emerged.

One reason for performing this analysis is that the complex composition of parasite ESP (hundreds of proteins in some cases) confounds the ability to identify those related to virulence and hence the success of an infection. Comparative analysis between ecologically diverse species may help prioritize proteins that play essential roles in parasitism for functional studies and may contribute to our understanding of the molecular basis of host-parasite specificity. The orthology analysis allowed to re-analyze all collected data simultaneously with the same tool, and resulted in a sequence-based clustering, independently of their initial annotation, reported in individual papers.

A number of classical protein signatures among parasitic nematode secretomes were previously identified. These include cytokine homologues, C-type lectins and galectins, protease inhibitors, antioxidants, and venom allergen homologues [9]. In addition, proteins involved in cell wall degradation, detoxification and giant cell formation, among others, are secreted by the plant parasite *M*. *incognita* [16].

We included the model organism *C*. *elegans*, as this highly studied, free-living nematode has been a powerful model in comparative genomics and proteomics studies [77]. Comparative analyses with a free-living species may reveal mechanisms by which parasitic species have arisen and evolved [78]. Indeed, our comparative analyses of secretomes has shown some differences between parasitic species and *C*. *elegans*, which for instance, was not found to secrete aldolase or enolase, peroxiredoxin/AhpC/TSA family, or galectin.

Transthyretin-like proteins were identified in all adult secretions by both Pfam and orthology analyses, while cyclophilin-type peptidyl-prolyl cis-trans isomerases were absent only from *N*. *americanus* ESP. In vertebrates, transthyretin-related proteins are transporters of thyroid hormones and retinoids, while putative functions vary in invertebrates [79]. Transthyretin was hypothesized to be secreted by *M*. *incognita* to regulate host plant cell growth, contribute to establishment of infection and participate in immune evasion [16]. In the parasitic nematode *Ostertagia ostertagi*, however, secondary structure predictions do not support the ability to achieve the conformation of transthyretin-like proteins necessary to transport lipids or thyroid hormones, which was confirmed in assays using recombinant protein [80]. Neuronal expression with functions in the nervous system was suggested for *C*. *elegans*, as transthyretin-like proteins, which show characteristics similar to neuropeptides [81]. Nematodes must acquire essential compounds such as fatty acids and retinol from their environment, as they are incapable of their *de novo* synthesis. Fatty acid and retinol binding (FAR) proteins were common across the adult secretomes of 16/18 species reviewed here. These highly conserved proteins may play a critical role in the sequestration of a broad range of essential fatty acids, retinoids and other nutrients for delivery from the host to the parasite [82]. Many parasites have limited capacity to synthesize lipids and therefore depend on lipid binding proteins to acquire essential metabolites from their host [83]. Hydrophobic lipophilic molecules such as fatty acids, eicosanoids, retinoids and steroids have important functions as energy sources and in metabolic signaling [83]. Nematode polyprotein antigen represents another class of fatty-acid binding proteins. In hookworms, it localizes in the subcuticle, suggesting a direct interaction with the host at the attachment site, although its precise role remains obscure [84].

### Missing proteins in *C*. *elegans* ESP

Absent from *C*. *elegans* ESP, several protein families may be specifically associated with parasitism, in agreement with the orthology analysis; they include the glycolytic enzymes enolase and aldolase, a peroxiredoxin, and an alkyl hydroperoxide reductase (AhpC) and thiol specific antioxidant (TSA). This is in line with recent data on the contents of *C*. *elegans* extracellular vesicles (EVs), in which these proteins were also absent [85], while enolase but not aldolase were described in EVs of several nematode species [33]. Together with GAPDH, and triosephosphate isomerase (TPI), enolase and aldolase from other pathogens can bind plasminogen, favoring extracellular matrix degradation, a phenomenon that favors host invasion [86,87]. Additional non-metabolic (moonlighting) functions have been attributed to aldolase and enolase [88,89]. For instance, their extracellular presence has been suggested to increase virulence of various pathogens (reviewed in [89]) by facilitating adherence to cells to invade, modulating host immune responses (especially by macrophages), interfering with the host hemostatic system, and stimulating angiogenesis around parasite structures to increase blood supply, among other speculative functions. For instance, GAPDH has anti-inflammatory properties, preventing cytokine storm and mortality in a mouse model of LPS-induced sepsis [90]. In T cells, GAPDH acts as an energy sensor post-transcriptionally; when glucose levels are low, its interaction with transcripts of key genes, including IFN-γ and IL-2, represses translation, restricting cytokine production under conditions of limiting energy resources [88,91]. In *H*. *contortus*, GAPDH has also been shown to bind to and inhibit the activity of complement C1q and C3, suggesting a role in host immunomodulation [92,93]. Plant parasitic nematode GAPDH has protective properties against ROS [16,94]. In humans, α-enolase can be translated as a truncated version called Myc-promoter-binding protein 1 (MBP-1), which represses the pro-proliferative promoter c-myc, governing metabolic reprogramming of T cells [95,96]. MBP-1 also suppresses inflammation through transcriptional potentiation of a FoxP3 isoform in regulatory T cells [90,97]. It is striking that aldolase, enolase and TPI were not detected in the secretome of *C*. *elegans*, while GAPDH is present. To conclude whether the secretion of glycolytic enzymes reflects an adaptation to a parasitic lifestyle will require further evidence based on secretome analysis from additional non-parasitic species. At this stage, we cannot exclude the possibility that the absence of these ESP represents a *C*. *elegans*-specific trait. In addition, whether these enzymes are active in the extracellular milieu remains to be determined.

Gene duplication is a major driver of functional diversification of proteins. This is particularly relevant in the context of parasitism, where an evolutionary arms race between host and parasite is in place, and with events such as host range expansion. In parasitic nematodes, it is likely that rapid gene family expansions occur frequently, along with switches in secretory capacity, to facilitate adaptation to specific niches [98]. Consistently, unique modifications of effectors may be observed in each taxa of closely related species [98]. For helminth proteases, it was hypothesized that evolution in protease gene families is rather driven by accidental fortuitous interactions with off-target substrates, than by gradual evolution. The acquisition of new functions would then be followed by multiple gene duplication events [98]. Adaptation to host metabolism has resulted in a loss of metabolic pathways in trematode and cestode parasites, but not in nematodes. Instead, nematodes have acquired additional enzymes via horizontal gene transfer, although the general trend in helminths is toward a reduction of auxiliary metabolism [98]. Upon phylogenetic analysis of the glycolytic enzymes aldolase and enolase, we found the expected divergence based on species, but also appreciable gene duplication (especially for aldolase), while the functional consequences of this multiplicity remain to be studied. Possible functions of ESP may be (at least partially) characterized by examining life stage-specific production patterns, which was not systematically possible in the present work. As several of the examined species have independent origins of parasitism, a systematic convergent evolution of an essential gene is unlikely to happen. Rather, some proteins present across eukaryotes might show similar moonlighting functions, and some organisms might have lost the ability to release these molecules.

Glycolytic enzymes lacking signal peptides, the mechanisms by which these moelcules are exported to the extracellular space are not completely resolved. However, they have been detected in EVs of various origins, also from helminths [33].

### Antioxidants and redox proteins

Reactive oxygen species (ROS) and redox signals functionally regulate various aspects of host-pathogen interactions, from pathogen entry through protein redox switches and redox modification, to phagocyte ROS production and control of phagolysosome function (and hence antigen processing) [99]. Parasites must counter the damaging effects of ROS released by stimulated host immune cells. Accordingly, several major antioxidant proteins were present in the analyzed secretomes. Although absent in the secretomes from *C*. *elegans*, *B*. *malayi*, and *S*. *venezuelensis* adults in this comparative analysis, glutathione S-transferase (GST) was described (although perhaps not secreted) for the free-living nematode in response to oxidative stress in general and nutrient deprivation [100,101], and in filarial parasites including *O*. *volvulus* [102]. In a *B*. *malayi* secretome as well, a GST had been reported, which has however been assigned to another orthogroup in the present analysis; at the time of writing, this sequence was no longer annotated as a GST, but may have GST-like activity. GST-like proteins appear to fulfil other functions, including immunomodulation. OvGST2 may protect the parasite from host-mediated lipid peroxidation, while OvGST1 was shown to generate eicosanoids, the anti-inflammatory properties of which may influence dendritic cell migration or chemotaxis of different cell types [53,103,104]. Similarly, superoxide dismutase appeared in 15 adult secretomes, and is likely to be common to parasitic nematodes [105]. Peroxiredoxins were detected in 16 secretomes and elsewhere in the secretions or tissues from *D*. *immitis*, *A*. *ceylanicum*, and *H*. *contortus* [105–108].

Protein disulfide isomerase (in 15/18 secretomes) belongs to a redox chaperone family with implications in many cellular processes. It participates in the maintenance of stable protein conformations. Host-derived protein disulfide isomerase determines the stability of antigen-MHC class I complexes and their transport to the plasma membrane, inhibits protozoan parasite uptake by immune cells via redox mechanisms, and modulates their translocation to phagosomes and ROS production by NADPH oxidase [99].

### Other common ESP

Peptidyl-prolyl cis-trans-isomerases (also known as cyclophilins), present in 17/18 secretomes and absent from *N*. *amercicanus*, function in extracellular signaling. For instance, some secreted cyclophilins induce chemotaxis or integrin-mediated immune cell adhesion [109].

Nucleoside diphosphate kinase, in addition to functions in the maintenance of intracellular nucleotide pools, serves as a mechanism in *Mycobacterium tuberculosis* to evade the innate immune system by promoting phagosome maturation arrest in macrophages and by inactivating small GTPases necessary for the production of ROS [110].

Over 40% of ESP released by plant parasites were ribosomal proteins. If their extracellular function(s) is/are unclear to date, targeting them by RNAi often results in a significant decrease in worm viability [111].

### Considerations on mechanisms of protein secretion

Reported proportions of ESP containing a signal peptide for classical secretion vary from 15 to 78% among parasitic species, and reached 93.5% in the *C*. *elegans* secretome (Table 1). Hypotheses to explain the perhaps surprisingly low rates in parasites include that the prediction algorithms were trained on mammalian protein sequences [112], or that holocrine secretion may occur [51]. Among known alternative mechanisms of secretion by parasites is the exosomal pathway; thus, absence of a signal peptide does not accurately predict the secretory status of a protein. According to the Exocarta database [113], the four glycolytic enzymes previously mentioned, actin, heat shock proteins, peptidyl-prolyl cis-trans isomerase, 14-3-3 proteins, some galactoside-binding lectins, elongation factors, and peroxiredoxins are among the 100 most often reported exosomal proteins in mammalian exosomes. Because Exocarta is based on mammalian data, it is not possible to confidently assign nematode proteins to the exosome compartment based on sequence alone. However, some of the most common proteins in nematode secretomes have been shown to be released via exosomes into the environment. For instance, in *T*. *muris* secretions, transthyretin was found in both “classical” ES and in the exosome fraction, while superoxide dismutase was found in exosomes only [57]. In helminth parasites, proteins bearing signal peptides are less evolutionarily conserved and and were proposed to undergo accelerated evolution, either because of loosened functional constraints, or as a consequence of a stronger selective pressure from the host immune system [114].

### Conclusions and priorities for further research

The identification of individual secretome components as potentially key determinants that render a host permissive to infection will expand the list of candidate targets that could be exploited for therapeutic intervention. Therefore, pinpointing elements that may have evolved as to enable parasitism is inherently useful.

Our comparative analysis workflow allowed to identify the most conserved ES proteins and protein families across 20 nematode secretomes (including 18 adult secretomes). Similarly, we identified two glycolytic proteins that were absent in the non-parasitic species *C*. *elegans*. A next step will be to validate this result in further non-parasitic nematode species. Defining potential implications of aldolase and enolase in parasitic processes should be a research priority. Further in-depth characterization of which moonlighting functions may be fulfilled by these and by which isoforms may shed light on novel host modulatory mechanisms.

## Supporting information

S1 Table Analyzed *C*. *elegans* secretome. (XLSX)

S2 Table Pfam accession analysis. (XLSX)
